# Mechanism of the Transmetalation of Organosilanes to Gold

**DOI:** 10.1002/open.201500172

**Published:** 2015-09-10

**Authors:** Laura Falivene, David J. Nelson, Stéphanie Dupuy, Steven P. Nolan, Albert Poater, Luigi Cavallo

**Affiliations:** ^1^ KAUST Catalysis CenterPhysical Sciences and Engineering DivisionKing Abdullah University of Science and TechnologyThuwal23955-6900Saudi Arabia; ^2^ WestCHEM Department of Pure & Applied ChemistryUniversity of StrathclydeThomas Graham Building295 Cathedral StreetGlasgowG1 1XLUK; ^3^ EaStCHEM School of ChemistryUniversity of St. AndrewsNorth HaughSt. AndrewsFifeKY16 9STUK; ^4^ Chemistry DepartmentCollege of ScienceKing Saud UniversityP.O. Box 2455Riyadh11451Saudi Arabia; ^5^ Institut de Química Computacional i Catàlisi and Departament de QuímicaUniversitat de GironaCampus Montilivi17071GironaCataloniaSpain

**Keywords:** DFT calculations, fluoride-free, gold catalysis, homogeneous catalysis, organosilanes, transmetalation

## Abstract

Density functional theory (DFT) calculations were carried out to study the reaction mechanism of the first transmetalation of organosilanes to gold as a cheap fluoride‐free process. The versatile gold(I) complex [Au(OH)(IPr)] permits very straightforward access to a series of aryl‐, vinyl‐, and alkylgold silanolates by reaction with the appropriate silane reagent. These silanolate compounds are key intermediates in a fluoride‐free process that results in the net transmetalation of organosilanes to gold, rather than the classic activation of silanes as silicates using external fluoride sources. However, here we propose that the gold silanolate is not the active species (as proposed during experimental studies) but is, in fact, a resting state during the transmetalation process, as a concerted step is preferred.

## Introduction

Thanks to the “gold rush” of the last two decades,^1^, ^2^ gold catalysis^1^ has enabled a vast range of exciting and useful transformations of organic molecules.^3^, ^4^ Homogeneous gold catalysis is most often mediated by gold(I) complexes bearing *N*‐heterocyclic carbene (NHC) or phosphine ligands,^5^ which can exhibit various modes of reactivity such as the activation of π‐acids^6^ or complex mechanistic pathways involving carbene species.^7^ More recently, the involvement of multiple gold centers has been shown to allow new and interesting transformations.^8^

**Scheme 1:**
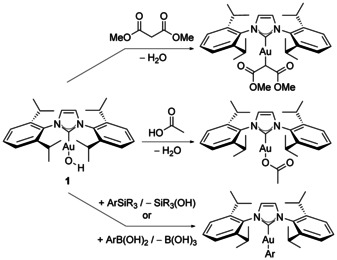
Reactions involving **1**.

Nolan and co‐workers have contributed to this area by developing the chemistry of gold(I) hydroxide complexes.^9^ These complexes can be activated by acid to form active “ [Au(NHC)]^+^ ” complexes without the need for silver salts,^10^ or can effect the activation of a range of suitable C−H/O−H bonds (where pK_a_<30.3)^11^ and also C−C bonds.^12^ Upon reaction with boronic acids and silanes, transmetalation results, providing new (typically air‐stable) organogold(I) complexes (Scheme 1 illustrates some of these pathways with [Au(OH)(IPr)] (**1**)).^13^, ^14^ These complexes therefore hold promise for the development of useful and selective catalytic transformations, as mechanistic probes, and can allow reactions to proceed through mechanisms other than the classical activation of π‐acid substrates. Understanding the reactivity of this class of complexes is critically important, as it allows us to fully exploit it in catalysis and in organic synthesis.

Transmetalation of a group from one metal (or metalloid) species to another is a key step in metal‐catalyzed crosscoupling reactions. Each example is distinct and can proceed through a different mechanism. For example there has been some debate recently regarding whether transmetalation in Suzuki–Miyaura reactions^15^ occurs via an organopalladium(II) halide and a boronate molecule, or via an organopalladium(II) hydroxide and a boronic acid.^16^ Few intermediates en route to transmetalation have been isolated and fully characterized. In this field, transmetalation to gold is of current interest,^9^, ^17^, ^18^ due to the potential to develop dual catalysis with gold and palladium, nickel, or rhodium, for example.^19^

**Scheme 2:**
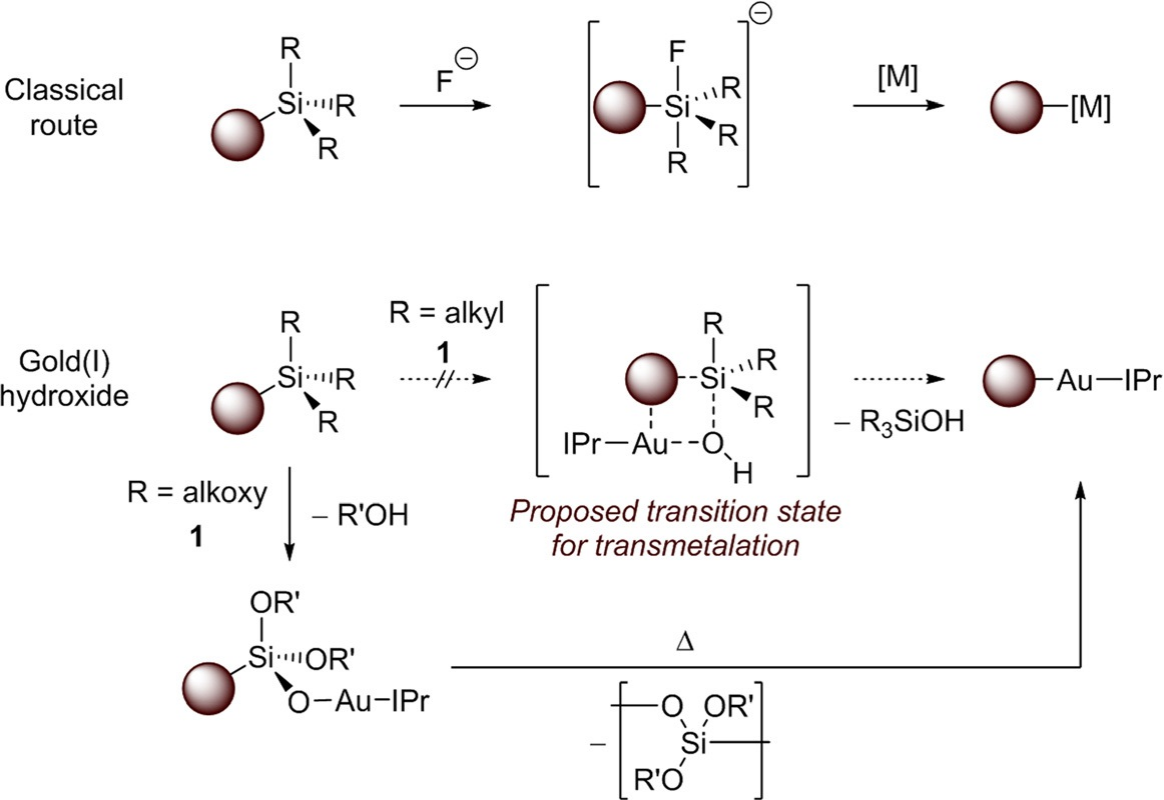
Classical fluoride‐activated transmetalation reaction pathway and the fluoride‐free route involving **1**.

Nolan and Dupuy have recently studied the transmetalation of silanes with gold(I) hydroxide complexes.^20^ In contrast to traditional transmetalation pathways involving silanes (e.g. the Hiyama cross‐coupling),^21^ no anionic fluoride was necessary to promote the reaction (Scheme 2). While reactions with simple silanes such as PhSiMe_3_ led to no product, silanolate species **2** were formed rapidly when **1** was exposed to aryltrialkoxysilanes, and these then evolved to the desired arylgold(I) species **3** on heating. Three possible mechanistic hypotheses were put forward: 1) electrophilic aromatic substitution to form a Wheland intermediate, followed by the loss of a silicate leaving group, 2) a concerted mechanism, proceeding through a four‐membered transition state, or 3) activation of the silane as a silicate, followed by a concerted transmetalation.

Interestingly, experimentally, this reaction was found to proceed far more rapidly when **1** was exposed to silane, compared with starting from isolated gold silanoate **2**, which stalled after 50 % conversion. For both reactions, the formation of pink particles was observed over time implying decomposition of the gold complex to gold(0) nanoparticles. These findings strongly emphasized the non‐innocent role of the molecule of methanol generated in situ when forming intermediate **2** before the subsequent transmetalation reaction. Certain of the benefits of methanol on reactivity, both reactions were repeated in methanol at 80 °C. Alas, no trace of **3** could be detected after 24 h. However, the addition of increased amounts of dry methanol, under these reaction conditions, proved to effectively enhance the overall rate of the transmetalation.

To shed light on the mechanism of the transmetalation of organosilanes to gold, density functional theory (DFT) calculations were performed, as these allow a greater depth of insight into the individual steps of this fluoride‐free transmetalation than experimental results.

## Results and Discussion

**Scheme 3:**
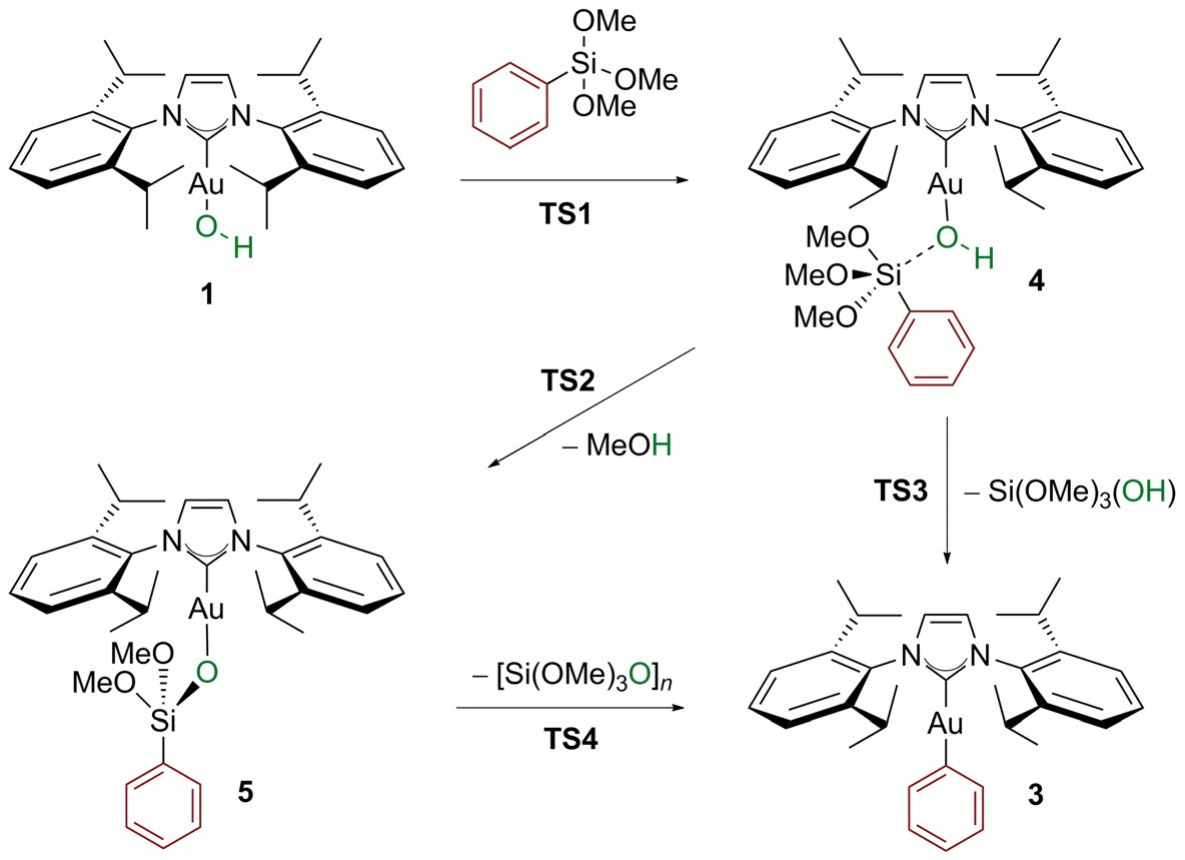
Calculated pathways for the transmetalation of silanes to gold(I) hydroxide **1**.

**Figure 1:**
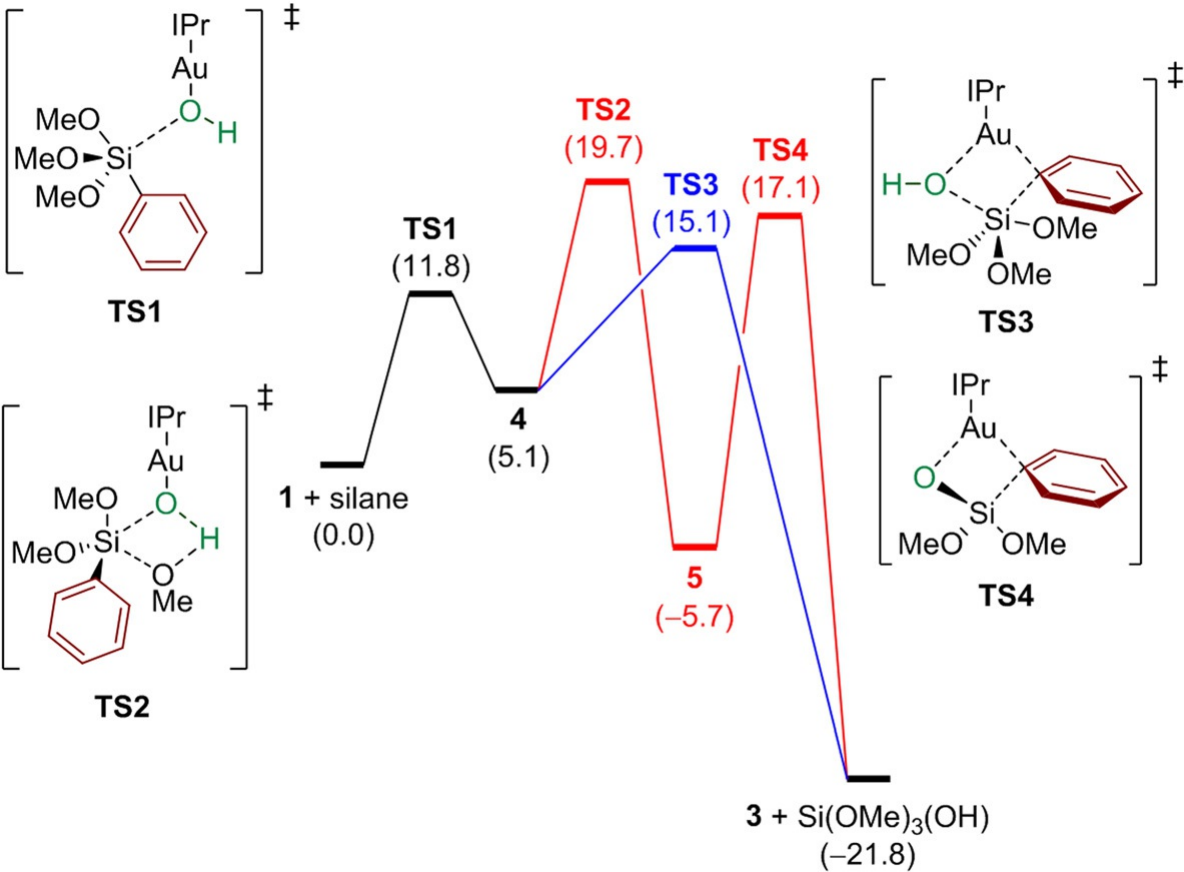
Free energy profile for the transmetalation of trimethylphenylsiloxane to [Au(OH)(IPr)] (**1**) (energies in kcal mol^−1^).

The reaction pathways that best link the organosilane reactant to the organogold product are displayed in Scheme 3, bearing a fluoride‐free route. The first common step highlighted by calculations of the reaction of trimethoxyphenylsilane with **1** leads to a weakly‐bound species **4** which holds a pentacoordinate silicon centre. The barrier to this intermediate (**TS1**) is only 11.8 kcal mol^−1^ (Figure 1), with the adduct lying 5.1 kcal mol^−1^ higher in energy than the starting materials. This is indicative of a plausible equilibrium in solution (*K*_eq_≈10^−4^ L mol^−1^).

Intermediate **4** undergoes to a proton transfer from the Au−OH moiety to one of the OMe groups of the silane resulting in the elimination of methanol with formation of product **5**. The energy barrier for this process is 14.6 kcal mol^−1^. Even though this barrier is rather high in comparison with experimental findings,^20^ it is still achievable at room temperature (**TS2** in Figure 1). Gold siloxane **5** and methanol are 5.7 kcal mol^−1^ more stable than **1** plus silane.

**Scheme 4:**
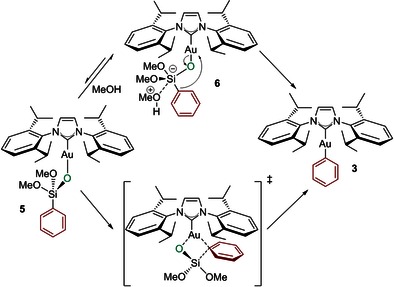
Proposed reaction pathways for the aryl transfer from the siloxane to gold(I).

From this point forward, two mechanisms were sought through which **5** could evolve into phenyl gold **3** and a silicon byproduct, which are placed 21.8 kcal mol^−1^ below **1** plus silane. With regard to preliminary experiments,^20^ and similar to Denmark's work,^22^ the pathways envisioned for the transmetalation from silicon to gold (Scheme 4) are: 1) an anionic pathway whereby the molecule of methanol, generated in situ, would act as a nucleophilic activator, generating a pentacoordinated gold silicate species **6** which is poised for transmetalation and 2) a thermal pathway that would proceed via a concerted mechanism through **TS4**. The latter pathway would most likely be more challenging and thus may rationalize the difference in reactivity when using a nonpolar solvent such as toluene versus 1,4‐dioxane.

**Figure 2:**
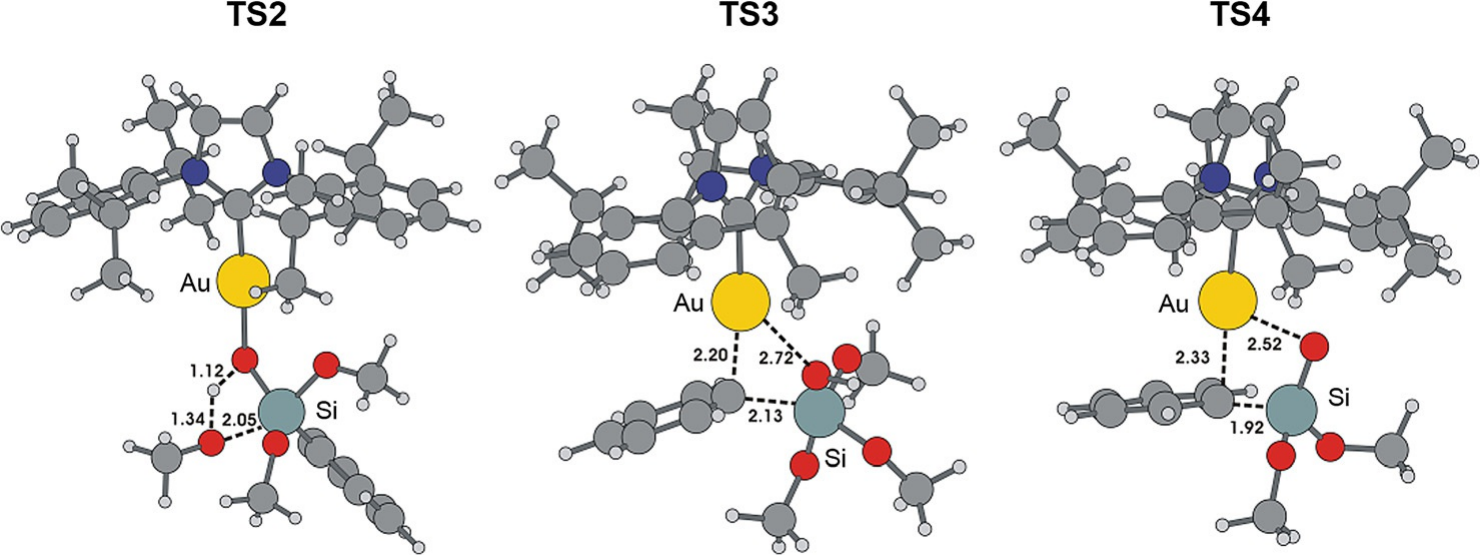
Located transition states **TS2**, **TS3**, and **TS4** in the transmetalation from phenyltrimethoxysilane to gold (selected distances in Å).

Attempts to locate the proposed intermediate **6** were unsuccessful. The most feasible pathway found was through transition state **TS4**, which releases a high energy Si(O)(OMe)_2_ molecule that can react in a barrierless step with a methanol molecule leading to the final gold complex **3** and Si(OMe)_3_OH. **TS4** lies 17.1 kcal mol^−1^ above **1** plus silane and more importantly, 22.8 kcal mol^−1^ above **5**, from which it defines the upper energy barrier in Figure 1. Finally, an alternative reaction involving the direct activation of the Si−Ph bond in **4** by the Au−OH bond in a single concerted step was explored. In this context, transition state **TS3** was located, 15.1 kcal mol^−1^ above **1** plus silane (10.0 kcal mol^−1^ above **4**), which connects intermediate **4** to the final products in a single step (Figure 1). All transition states are represented in Figure 2.

The overall mechanistic scenario emerging from Figure 1 is rather complicated. These computational results point out that the formation of gold adduct **4** through **TS1** is feasible kinetically, and this intermediate can be considered as the key intermediate in the reaction. Two alternative pathways were located for the transmetalation reaction to proceed from **4**. From **4**, the reaction could go through **TS2** to give **5** and then via a four‐membered transition state **TS4** to eliminate a silicon byproduct and produce **3**. Alternatively, the second possibility is the direct transmetalation process from **4** through **TS3** leading to phenyl gold complex **3**. Considering the difference in energy of 4.6 kcal mol^−1^ between the determining transition states of the two pathways, the latter is likely to be favored.

In the context of the experimental results, this potential energy surface is sensible. While the mixing of **1** and silane has been shown to yield isolable species such as **5**, the isolated complex **5** is known to undergo transmetalation much slower than the combination of **1** and silane. While this was interpreted as implying a special role for methanol, liberated during this first step, in the subsequent transmetalation step, it could instead point to the reversible formation of **5**, from the computational results. In fact, complex **5** faces a 25.4 kcal mol^−1^ barrier to reform species **4**, which may be surmounted under the high temperature conditions used, despite a 22.8 kcal mol^−1^ barrier to transmetalation. In contrast, **4** faces only a 10.0 kcal mol^−1^ barrier to direct transmetalation. We therefore propose that, in solution, **5** is a resting state lying off the transmetalation pathway in a potential energy well, and that it is not a true intermediate in the reaction; it must first be (re)converted to **4**.

## Conclusions

We have shed light upon this new synthetic approach to NHC‐aryl‐, vinyl‐, and allyl‐gold systems. This is the first computational study of a transmetalation of organosilanes to gold under fluoride‐free conditions, representing a different reactivity manifold than the classic activation of silanes as silicates using external fluoride sources.^23^ The present results provide the first key insight into the mechanism of transfer of the organic fragment from silane to gold and establishes that the reactivity of gold is similar to that of palladium in the Hiyama‐type coupling. We propose that the gold silanolate is not the active species (as proposed during experimental studies) but is, in fact, a resting state during the transmetalation process, as a concerted step is preferred. The potential energy surface in Figure 1 explains why isolated **5** undergoes aryl transfer to gold much slower than mixing **1** and silane directly. The basic understanding of this transmetalation reaction of silanes lays the groundwork for further exciting studies in this area

## Computational Details

All DFT calculations were completed with the Gaussian09 set of programs.^24^ For geometry optimizations, the well‐established and computationally fast generalized gradient approximation (GGA) functional BP86 was used.^25^ Geometry optimizations were performed without symmetry constraints, while located stationary points were characterized by analytical frequency calculations. The electronic configuration of the molecular systems was described with the split valence polarized (SVP) basis set with a polarization function for H, C, N, Si, and O.^26^ For Au, we used the small‐core, quasi‐relativistic Stuttgart/Dresden effective core potential with an associated valence contracted basis set (standard SDD keywords in Gaussian 09).^27^ Zero‐point energies and thermal corrections were calculated at the BP86 level. Single‐point energy calculations with the M06 functional^28^ in solution were performed with the triple‐zeta valence with polarization (TZVP) basis set for main group atoms and again the same SDD pseudopotential for Au. Solvent effects were included with the polarizable continuous solvation model (PCM) using 1,4‐dioxane as a solvent.^29^ The reported free energies in this work include energies obtained at the M06/TZVP level corrected with zero‐point energies, thermal corrections, and entropy effects evaluated at 298 K and 1354 atm^30^ with the BP86/SVP method in the gas phase.

## Supporting information

As a service to our authors and readers, this journal provides supporting information supplied by the authors. Such materials are peer reviewed and may be re‐organized for online delivery, but are not copy‐edited or typeset. Technical support issues arising from supporting information (other than missing files) should be addressed to the authors.

Supplementary
