# Supplementary material for: Mechanism of the Transmetalation of Organosilanes to Gold
Source: ChemistryOpen. 2015 Sep 10;5(1):60–4. doi: 10.1002/open.201500172 (PMC4906480; doi:10.1002/open.201500172)
Supplement: Supplementary file 1 — Supplementary [file OPEN-5-60-s001.pdf]

ChemistryOPEN

Supporting Information

© 2016 The Authors. Published by Wiley-VCH Verlag GmbH & Co. KGaA, Weinheim

Mechanism of the Transmetalation of Organosilanes to Gold

Laura Falivene,[a] David J. Nelson,[b] Stéphanie Dupuy,[c] Steven P. Nolan,*[c, d] Albert Poater,*[e] and Luigi Cavallo*[a]

open_201500172_sm_miscellaneous_information.pdf

Supporting Information of:

Mechanistic Insights on the Transmetalation of Organosilanes to Gold

Laura Falivene, David J. Nelson, Stéphanie Dupuy, Steven P. Nolan,* Albert Poater,* and Luigi Cavallo*

Computational details **S1**
Cartesian coordinates with solvent-phase free energies **S1–S17**
3D structures **S17–S18**

Computational details

All DFT calculations were completed with the Gaussian09 set of programs.[i] For geometry optimizations, the well-established and computationally fast GGA functional BP86 was used.[ii] Geometry optimizations were performed without symmetry constraints, while located stationary points were characterized by analytical frequency calculations. The electronic configuration of the molecular systems was described with the SVP basis set with a polarization function for H, C, N, Si, and O.[iii] For Au, we used the small-core, quasi-relativistic Stuttgart/Dresden effective core potential with an associated valence contracted basis set (standard SDD keywords in Gaussian 09).[iv] Zero point energies and thermal corrections were calculated at the BP86 level. Single-point energy calculations with the BP86 functional in solution were performed with the TZVP basis set for main group atoms and again the same SDD pseudopotential for Au. Solvent effects were included with the polarizable continuous solvation model PCM using 1,4-dioxane as a solvent.[V] Reported energies are M06/TZVP~SDD//BP86/SVP~SDD electronic energies corrected with ZPEs, thermal energies, and entropy effects calculated at 298 K and 1354 atm using the BP86/SVP~SDD method. We also corrected the energy for solvation effects present in an 1,4-dioxane solution that were calculated at the M06/TZVP~SDD//BP86/SVP~SDD level with the PCM method.

Cartesian coordinates with solvent-phase free energies (in a.u.)

1

E =-1370.64175232 A.U.

C	3.130095	1.263013	-0.641050
C	2.463038	0.024496	-0.834671
C	3.104214	-1.228086	-0.645423
C	4.460258	-1.211171	-0.258986
C	5.146532	-0.004068	-0.068187

C	4.485567	1.217741	-0.255643
N	1.077843	0.041685	-1.256056
C	-0.006843	0.023328	-0.403685
N	-1.098796	0.054815	-1.245489
C	-0.701090	0.092074	-2.583357
C	0.668737	0.083536	-2.589728
Au	0.017848	-0.063147	1.568754
O	0.150209	-0.185541	3.561752
H	-0.758639	-0.189010	3.923160
C	-2.481703	0.043754	-0.818009
C	-3.132653	1.283693	-0.583211
C	-4.489717	1.243343	-0.202054
C	-5.167830	0.024798	-0.059027
C	-4.497118	-1.184335	-0.289391
C	-3.140334	-1.205775	-0.673223
C	-2.406843	2.624936	-0.686087
C	-2.219322	3.249864	0.714217
C	-2.424260	-2.541652	-0.872508
C	-2.256433	-3.276360	0.475969
C	2.374508	-2.561494	-0.807474
C	2.224250	-3.274781	0.554493
C	2.422698	2.609901	-0.790366
C	2.251682	3.291321	0.585430
C	-3.109672	3.596828	-1.656447
C	-3.125794	-3.429712	-1.921344
C	3.053824	-3.470355	-1.853602
C	3.134580	3.534134	-1.800121
H	-5.022579	2.187652	-0.007858
H	-5.035886	-2.136683	-0.163120
H	4.985225	-2.165776	-0.096571
H	5.030279	2.160782	-0.090665
H	-6.227914	0.017234	0.240826
H	6.204899	-0.015503	0.237334
H	-1.405191	-2.323228	-1.254486
H	-2.552202	-4.366821	-2.080786
H	-4.147508	-3.721126	-1.597893
H	-3.218364	-2.915018	-2.900346
H	-1.682356	-4.216835	0.338372
H	-1.713388	-2.644074	1.209234
H	-3.240912	-3.543981	0.915609
H	-1.393088	2.428673	-1.093186
H	-1.636466	4.192539	0.645845
H	-3.197161	3.491422	1.183001
H	-1.676773	2.555415	1.389687
H	1.350812	-2.341823	-1.176248
H	2.473894	-4.407627	-1.987523
H	3.132289	-2.972500	-2.842526
H	4.079736	-3.759949	-1.542029
H	1.643532	-4.214216	0.438696
H	3.213609	-3.542102	0.982895

H	1.697378	-2.630231	1.288988
H	1.404197	2.413319	-1.185678
H	1.682168	4.239138	0.482514
H	1.705353	2.632427	1.292029
H	3.234950	3.537169	1.039979
H	2.566347	4.479365	-1.927953
H	4.155373	3.808144	-1.459193
H	3.231714	3.056051	-2.797107
H	-2.525342	4.535627	-1.755435
H	-3.222328	3.156014	-2.668842
H	-4.122896	3.876994	-1.298279
H	1.387296	0.102721	-3.414836
H	-1.427280	0.119442	-3.401453

PhSi(OMe)3

E= -1370.64175232 A.U.

C	-1.164756	-1.218507	2.406971
O	-1.462670	-1.149294	1.021031
Si	-0.917163	0.035699	-0.033807
C	0.954503	0.112037	-0.034155
O	-1.613030	-0.356309	-1.498955
C	-1.675831	-1.646185	-2.084608
O	-1.434004	1.577008	0.328917
C	-2.744408	2.085546	0.112589
H	-1.660314	-2.117508	2.830247
H	-0.070117	-1.304603	2.598999
H	-1.539137	-0.325910	2.958040
H	-2.388572	-1.613573	-2.935568
H	-0.684840	-1.966891	-2.482196
H	-2.027998	-2.414531	-1.360536
H	-2.691383	3.193978	0.076747
H	-3.176227	1.719877	-0.844645
H	-3.433280	1.799767	0.939679
C	1.636682	1.344253	-0.182318
C	3.040589	1.391969	-0.216061
C	3.789646	0.206866	-0.101612
C	3.130523	-1.025717	0.051503
C	1.725800	-1.070531	0.088498
H	1.052405	2.274451	-0.265653
H	3.554986	2.359913	-0.330917
H	4.890733	0.244361	-0.128658
H	3.714020	-1.955809	0.146583
H	1.221155	-2.042935	0.218393

4

E= -2236.91763273 A.U.

C	-1.732390	0.617042	0.203883
N	-1.850027	1.927489	0.603382
N	-3.012528	0.118057	0.269132
C	-3.174439	2.236134	0.911933

C	-3.906913	1.096810	0.701347
C	-0.758683	2.877376	0.690250
C	-3.395338	-1.236904	-0.073565
H	-3.470200	3.233597	1.250696
H	-4.975280	0.891148	0.817462
C	-0.417786	3.628842	-0.465147
C	0.621546	4.573113	-0.337513
C	1.288719	4.759634	0.880848
C	0.932447	3.998608	2.002240
C	-0.098404	3.038821	1.936241
C	-1.118972	3.443682	-1.811091
H	0.919011	5.169797	-1.214235
H	2.100946	5.499922	0.954387
H	1.470814	4.145808	2.951743
C	-0.447515	2.210257	3.172607
C	-3.370186	-2.228089	0.942157
C	-3.759604	-3.534460	0.580233
C	-4.156118	-3.837879	-0.729324
C	-4.171219	-2.836834	-1.710756
C	-3.793031	-1.512445	-1.408716
C	-2.911052	-1.935816	2.370744
H	-3.745607	-4.330602	1.341224
H	-4.453479	-4.866320	-0.989018
H	-4.479395	-3.088954	-2.737776
C	-3.788448	-0.449061	-2.507645
H	-1.876362	2.641455	-1.689072
C	-0.133229	2.968027	-2.899401
C	-1.868366	4.723308	-2.240875
H	-1.287826	1.536523	2.904037
C	0.736003	1.312271	3.590939
C	-0.928260	3.100317	4.338970
H	-2.737424	-0.842272	2.453008
C	-3.989614	-2.304346	3.411267
C	-1.566787	-2.634654	2.669407
H	-3.566556	0.528967	-2.031397
C	-2.662331	-0.720891	-3.529077
C	-5.162928	-0.318265	-3.196242
H	-0.670116	2.785343	-3.853989
H	0.653747	3.725381	-3.098570
H	0.370062	2.026384	-2.599117
H	-2.415425	4.553312	-3.192058
H	-2.605125	5.044559	-1.475377
H	-1.168616	5.569567	-2.407006
H	0.450834	0.674132	4.453762
H	1.053913	0.649710	2.760303
H	1.615054	1.916275	3.900396
H	-1.230326	2.476052	5.206025
H	-0.126130	3.784959	4.687349
H	-1.798190	3.725711	4.048523
H	-3.655146	-2.020699	4.431090

H	-4.950449	-1.786326	3.209944
H	-4.193292	-3.395885	3.424580
H	-1.219530	-2.384240	3.693736
H	-1.664715	-3.739487	2.610079
H	-0.777906	-2.322695	1.953539
H	-2.630991	0.079414	-4.297961
H	-1.672887	-0.757115	-3.026956
H	-2.816317	-1.687611	-4.053689
H	-5.145402	0.504489	-3.941360
H	-5.442826	-1.246132	-3.738203
H	-5.969213	-0.100805	-2.465229
Au	-0.106535	-0.332574	-0.382891
O	1.570411	-1.268353	-1.111693
H	1.362167	-2.106920	-1.578469
Si	3.139934	-1.846081	-0.067418
O	2.094719	-1.726295	1.292763
O	4.459987	-2.484033	0.886559
O	2.950533	-3.185918	-1.149221
C	4.024860	-0.270178	-0.689580
C	4.093847	0.063159	-2.063599
C	4.677304	0.593204	0.223348
C	4.788081	1.202135	-2.509297
H	3.588589	-0.583667	-2.798739
C	5.354164	1.747869	-0.211196
H	4.660900	0.353414	1.299817
C	5.416253	2.053515	-1.582254
H	4.835969	1.430523	-3.587313
H	5.844903	2.407706	0.523870
H	5.956122	2.950586	-1.928049
C	3.589120	-4.442185	-1.054874
H	3.963484	-4.646743	-0.028402
H	2.866060	-5.243182	-1.330938
H	4.451200	-4.514308	-1.759981
C	2.386586	-2.181409	2.601723
H	1.650013	-1.725889	3.300658
H	2.295578	-3.289354	2.687306
H	3.412983	-1.912265	2.930408
C	5.812474	-2.588458	0.532029
H	6.146634	-1.799027	-0.184134
H	6.450305	-2.494303	1.442878
H	6.056523	-3.577017	0.067521

5

E= -2121.27278238 A.U.

C	-4.452249	0.167502	-0.520555
C	-4.249188	-1.227951	-0.655781
C	-5.091760	-1.937851	-1.544680
C	-6.090331	-1.277392	-2.282021
C	-6.270929	0.110292	-2.139939
C	-5.450661	0.832444	-1.254493

Si	-2.870743	-2.088712	0.302541
O	-2.833059	-1.448604	1.876901
C	-3.953461	-1.512175	2.742606
O	-1.373275	-1.975299	-0.341801
Au	0.052217	-0.567354	-0.145135
C	1.496070	0.755774	0.042926
N	1.388442	2.093030	0.351215
C	2.646628	2.689373	0.434137
C	3.568138	1.709584	0.170509
N	2.851263	0.536724	-0.065205
C	0.144482	2.804623	0.562973
C	-0.408857	2.835269	1.869775
C	-1.596104	3.574343	2.052211
C	-2.206012	4.245228	0.983386
C	-1.643410	4.183872	-0.299193
C	-0.456360	3.462841	-0.542435
C	3.464095	-0.739745	-0.368087
C	3.702953	-1.074614	-1.726774
C	4.337467	-2.306620	-1.987952
C	4.714640	-3.164982	-0.945909
C	4.456442	-2.811206	0.385620
C	3.824796	-1.592541	0.708433
C	0.222220	2.101993	3.053094
C	0.758740	3.094892	4.107842
C	0.108560	3.378545	-1.960588
C	0.397944	4.772240	-2.556313
C	3.273982	-0.175706	-2.886678
C	4.470003	0.236448	-3.770632
C	3.531316	-1.249644	2.169245
C	4.821746	-1.181212	3.013581
C	-0.757868	1.085350	3.676655
C	-0.823164	2.549916	-2.872265
C	2.155166	-0.844513	-3.714886
C	2.506517	-2.233603	2.774719
O	-3.361047	-3.701014	0.346320
C	-2.493203	-4.785123	0.628433
H	-2.053637	3.616411	3.052955
H	-2.139882	4.701897	-1.134794
H	4.534656	-2.601106	-3.030844
H	4.746142	-3.498463	1.196335
H	-3.133907	4.815294	1.149698
H	5.208451	-4.122847	-1.174414
H	1.076815	2.838115	-1.907930
H	0.866624	4.675447	-3.558119
H	-0.530881	5.367542	-2.684858
H	1.085504	5.359480	-1.912545
H	-0.379751	2.438381	-3.884144
H	-0.994032	1.536260	-2.454734
H	-1.813575	3.037974	-2.990548
H	1.089362	1.526075	2.667791

H	-0.249792	0.505084	4.475482
H	-1.627391	1.595378	4.143715
H	-1.147170	0.369263	2.923036
H	2.847950	0.753432	-2.453720
H	4.141233	0.936815	-4.566825
H	5.263270	0.739458	-3.179317
H	4.931301	-0.640309	-4.272464
H	1.807052	-0.164723	-4.520888
H	2.511327	-1.780360	-4.195489
H	1.283459	-1.099335	-3.077190
H	3.067780	-0.241410	2.193402
H	2.258075	-1.942630	3.816994
H	1.565448	-2.244959	2.187082
H	2.905590	-3.269781	2.801913
H	4.588676	-0.873139	4.054473
H	5.329584	-2.167593	3.066861
H	5.547973	-0.453024	2.596094
H	-2.928401	-5.715163	0.201549
H	-1.482010	-4.635532	0.187975
H	-2.371544	-4.941392	1.726404
H	-3.647969	-1.157131	3.750768
H	-4.794258	-0.867079	2.392313
H	-4.347306	-2.549808	2.847162
H	-4.960074	-3.026816	-1.649993
H	-6.734720	-1.848723	-2.970561
H	-7.054214	0.628768	-2.717046
H	-5.591976	1.919234	-1.133736
H	-3.816556	0.742636	0.173928
H	1.253727	2.550289	4.939283
H	1.497951	3.799458	3.672615
H	-0.061422	3.701004	4.548389
H	4.661155	1.736768	0.128855
H	2.767667	3.750944	0.669974

3

E= -1526.27766062 A.U.

C	1.209035	3.875459	-0.240040
C	0.000487	3.128358	-0.192740
C	-1.207848	3.876255	-0.231891
C	-1.212291	5.281708	-0.310691
C	0.000886	5.991791	-0.353596
C	1.213862	5.280910	-0.318836
Au	0.000266	1.083656	-0.066871
C	-0.000104	-0.966988	0.071062
N	-1.084778	-1.811326	0.138617
C	-0.685832	-3.143990	0.247487
C	0.684916	-3.144221	0.247450
N	1.084294	-1.811686	0.138593
C	-2.469985	-1.389242	0.090881
C	-3.114060	-1.313504	-1.172136
C	-4.469851	-0.925708	-1.186257

C	-5.152873	-0.628732	0.001185
C	-4.489371	-0.708833	1.233272
C	-3.133694	-1.089855	1.309597
C	2.469626	-1.389974	0.091093
C	3.133566	-1.092082	1.310048
C	4.489340	-0.711349	1.233976
C	5.152703	-0.630087	0.001893
C	4.469462	-0.925623	-1.185785
C	3.113576	-1.313084	-1.171924
C	-2.385762	-1.590278	-2.488023
C	-3.100970	-2.661079	-3.338079
C	-2.425029	-1.127075	2.663455
C	-3.145830	-2.043127	3.674162
C	2.425002	-1.130464	2.663928
C	3.145916	-2.047253	3.673873
C	2.384946	-1.588099	-2.487990
C	3.100423	-2.656906	-3.340307
C	-2.176439	-0.280580	-3.279767
C	-2.237162	0.302651	3.216891
C	2.237054	0.298807	3.218538
C	2.174461	-0.277132	-3.277337
H	-4.998390	-0.849440	-2.149444
H	-5.032686	-0.463528	2.159561
H	5.032831	-0.467193	2.160463
H	4.997911	-0.848500	-2.148952
H	-6.211559	-0.326116	-0.034506
H	6.211462	-0.327703	-0.033604
H	1.407850	-3.963283	0.312174
H	-1.409040	-3.962810	0.312186
H	-1.411209	-1.549300	2.501848
H	-2.570542	-2.102029	4.621885
H	-4.157764	-1.662380	3.928013
H	-3.264496	-3.074559	3.281524
H	-1.671378	0.280551	4.172084
H	-1.677725	0.936309	2.496442
H	-3.215061	0.790850	3.415116
H	-1.379326	-1.987405	-2.239281
H	-1.603283	-0.474484	-4.210831
H	-3.147479	0.173957	-3.569801
H	-1.617151	0.465303	-2.676919
H	-2.512927	-2.884517	-4.252925
H	-3.234866	-3.609661	-2.777552
H	-4.105364	-2.322335	-3.669403
H	1.411191	-1.552619	2.502068
H	2.570672	-2.106975	4.621570
H	3.264687	-3.078361	3.280416
H	4.157817	-1.666609	3.928006
H	1.671432	0.275854	4.173812
H	3.214927	0.786958	3.416993
H	1.677458	0.932985	2.498694

H	1.378883	-1.986344	-2.239487
H	1.600992	-0.469733	-4.208481
H	1.614977	0.467370	-2.672918
H	3.145118	0.178472	-3.566991
H	2.512047	-2.879011	-4.255256
H	4.104423	-2.316940	-3.671566
H	3.235189	-3.606429	-2.781588
H	-2.175306	3.346458	-0.200486
H	-2.170822	5.827523	-0.338461
H	0.001038	7.092730	-0.414032
H	2.172538	5.826100	-0.353079
H	2.176349	3.345043	-0.215294

$Si(OMe)_3OH$

E= -710.68287536 A.U.

Si	-0.004759	-0.023382	0.310979
O	1.194320	-0.297744	-0.806023
O	-0.682890	1.438296	-0.091201
O	-1.252834	-1.120473	0.338426
O	0.713576	-0.099985	1.812859
H	0.096059	-0.326043	2.531410
C	2.213231	-1.282032	-0.692318
H	3.109441	-0.934371	-1.248661
H	2.503533	-1.459892	0.366652
H	1.887720	-2.250587	-1.135353
C	0.045858	2.603545	-0.451926
H	-0.667386	3.355442	-0.849814
H	0.564389	3.050601	0.426943
H	0.807756	2.388787	-1.233670
C	-2.298610	-1.188687	-0.622853
H	-3.130716	-1.787591	-0.196793
H	-2.685204	-0.179302	-0.885029
H	-1.959222	-1.687402	-1.559297

TS1

E= -2236.9069324 A. U.

C	4.649503	1.349350	0.044506
C	4.457222	0.161643	-0.698762
C	4.761649	0.174521	-2.079439
C	5.245608	1.339125	-2.698962
C	5.432399	2.513689	-1.947271
C	5.131584	2.516732	-0.573448
Si	3.917051	-1.405205	0.163467
O	2.906949	-1.189094	1.483989
C	3.093551	-1.740426	2.778649
O	1.694334	-0.568816	-1.149861
Au	-0.141679	-0.150397	-0.455333
C	-1.929758	0.368915	0.206281
N	-3.071177	-0.400799	0.280167
C	-4.144292	0.338505	0.780883
C	-3.673193	1.601153	1.028494

N	-2.323895	1.604768	0.673419
C	-3.161562	-1.793336	-0.105317
C	-2.924425	-2.787299	0.880128
C	-3.044266	-4.135463	0.484250
C	-3.387091	-4.477812	-0.831067
C	-3.613866	-3.474336	-1.783178
C	-3.507754	-2.109061	-1.445777
C	-1.465909	2.767391	0.771652
C	-0.778737	3.006246	1.990695
C	0.019461	4.166114	2.068982
C	0.133475	5.044450	0.982963
C	-0.548961	4.777287	-0.211804
C	-1.364444	3.635131	-0.347804
C	-2.511413	-2.442665	2.310772
C	-3.447664	-3.079166	3.358876
C	-3.723458	-1.037969	-2.514924
C	-5.093349	-1.175286	-3.211532
C	-0.867802	2.060499	3.188543
C	0.515656	1.482064	3.555380
C	-2.064560	3.352253	-1.677145
C	-2.961111	4.525319	-2.125179
C	-1.034702	-2.823024	2.556400
C	-2.562364	-1.041586	-3.533766
C	-1.537773	2.744093	4.400291
C	-1.036667	2.972065	-2.765661
O	5.289099	-2.059304	0.940711
C	6.560816	-2.190619	0.339918
O	3.467033	-2.588258	-0.964097
C	3.532412	-3.981239	-0.699803
H	-3.874978	-3.755266	-2.815573
H	-2.589325	-1.341330	2.426928
H	-0.717787	-2.518089	3.576010
H	-0.882962	-3.920208	2.470603
H	-3.161665	-2.753897	4.381128
H	-4.506663	-2.791337	3.192611
H	-3.394979	-4.188418	3.341125
H	-2.859626	-4.932363	1.221814
H	-3.474387	-5.537867	-1.118233
H	-2.699000	-0.234663	-4.284334
H	-3.710016	-0.050388	-2.008111
H	-5.929336	-1.158005	-2.481629
H	-5.247801	-0.341512	-3.928114
H	-5.171073	-2.121196	-3.788412
H	-2.509263	-2.006488	-4.081718
H	-1.587929	-0.880327	-3.026170
H	-0.366699	-2.324872	1.823105
H	-5.137348	-0.101549	0.913293
H	-4.169837	2.493688	1.420992
H	1.219537	2.278724	3.877447
H	0.972748	0.947777	2.697435

H	0.420037	0.764984	4.397966
H	-1.643893	2.028759	5.242984
H	-0.936461	3.602979	4.766926
H	-2.548637	3.127953	4.149325
H	-2.368034	5.440501	-2.335130
H	-3.501828	4.264150	-3.059102
H	-3.716102	4.784287	-1.353818
H	-2.724661	2.472072	-1.528771
H	-0.442212	5.466414	-1.064195
H	0.766920	5.941926	1.065679
H	-0.345150	3.814176	-2.980651
H	-0.424061	2.101558	-2.450333
H	-1.552594	2.709216	-3.713276
H	0.569254	4.379207	2.999458
H	1.735043	-1.490954	-1.480875
H	2.700609	-1.022094	3.530894
H	2.525420	-2.692679	2.893353
H	4.163391	-1.944252	2.997942
H	3.804235	-4.515916	-1.636049
H	4.290144	-4.225168	0.078213
H	2.545802	-4.371004	-0.358084
H	6.898282	-1.248944	-0.153441
H	7.305295	-2.450696	1.124153
H	6.585774	-2.999359	-0.429912
H	4.603388	-0.737657	-2.677411
H	5.474300	1.332864	-3.777393
H	5.810963	3.427815	-2.433292
H	5.271718	3.435062	0.020268
H	4.410551	1.365924	1.120699
H	-1.508280	1.203123	2.894472

TS2

E= -2236.89441531 A.U.

C	1.895121	0.467599	0.042210
N	3.103478	-0.188969	0.090480
N	2.225924	1.794255	-0.096644
C	4.163077	0.710901	-0.017564
C	3.610027	1.959290	-0.136670
C	3.270388	-1.622071	0.223120
C	1.288786	2.896969	-0.191729
H	5.205718	0.379464	-0.000883
H	4.068409	2.946803	-0.245207
C	3.371399	-2.405569	-0.956600
C	3.561220	-3.793937	-0.797031
C	3.646312	-4.373613	0.476364
C	3.540332	-3.573899	1.622681
C	3.351419	-2.179780	1.526372
C	3.244652	-1.808017	-2.358343
H	3.638435	-4.433833	-1.690180
H	3.792181	-5.460896	0.576628
H	3.600366	-4.042168	2.617822

C	3.201017	-1.340012	2.794999
C	0.930407	3.584129	0.997747
C	0.068864	4.692662	0.869880
C	-0.411384	5.097105	-0.383338
C	-0.053332	4.387239	-1.537527
C	0.801072	3.267118	-1.471959
C	1.394929	3.130496	2.381824
H	-0.236134	5.245501	1.772280
H	-1.082317	5.967411	-0.459609
H	-0.452976	4.702182	-2.514329
C	1.139294	2.490038	-2.743600
H	3.196552	-0.703961	-2.253303
C	1.926779	-2.256801	-3.027257
C	4.469377	-2.129533	-3.240070
H	3.162655	-0.272897	2.491192
C	1.866412	-1.659223	3.503885
C	4.403914	-1.499418	3.748261
H	2.161018	2.339378	2.239217
C	2.059682	4.268259	3.183562
C	0.221372	2.494357	3.160195
H	1.794537	1.641895	-2.454826
C	-0.133423	1.883887	-3.374594
C	1.922720	3.354804	-3.753991
H	1.812046	-1.779770	-4.023172
H	1.902861	-3.357224	-3.175468
H	1.049745	-1.976756	-2.407368
H	4.378157	-1.628954	-4.226734
H	5.415217	-1.788737	-2.769843
H	4.563905	-3.219206	-3.432057
H	1.731199	-1.008483	4.393113
H	1.005707	-1.497434	2.821694
H	1.834877	-2.714393	3.848915
H	4.292800	-0.826949	4.624490
H	4.486965	-2.535645	4.138906
H	5.362452	-1.251996	3.246587
H	2.439370	3.886580	4.154431
H	2.914068	4.714740	2.633500
H	1.343010	5.085107	3.411173
H	0.567184	2.107922	4.142088
H	-0.582596	3.235870	3.352203
H	-0.226998	1.653282	2.591787
H	0.133023	1.232484	-4.233302
H	-0.703663	1.279381	-2.639131
H	-0.812176	2.675292	-3.756946
H	2.192083	2.757552	-4.650441
H	1.320137	4.220441	-4.101860
H	2.860889	3.754925	-3.315674
Au	0.103275	-0.343483	0.126219
O	-1.720237	-1.274064	0.222953
H	-2.061410	-2.325156	0.055518

Si	-3.229325	-0.774924	-0.545781
O	-2.640256	0.858704	-0.579892
O	-3.732420	-0.757094	-2.173532
O	-3.184003	-2.825059	-0.484926
C	-4.666604	-0.723008	0.684718
C	-4.533369	-1.229745	2.000150
C	-5.907621	-0.141789	0.328837
C	-5.591512	-1.155803	2.921780
H	-3.581930	-1.693808	2.305976
C	-6.972472	-0.066655	1.246536
H	-6.053330	0.259554	-0.688110
C	-6.816830	-0.573830	2.547492
H	-5.462086	-1.558286	3.940287
H	-7.929416	0.389050	0.942295
H	-7.648688	-0.517807	3.268726
C	-2.935150	-3.712313	-1.548989
H	-3.882306	-4.138792	-1.955925
H	-2.393078	-3.242165	-2.408389
H	-2.318628	-4.575997	-1.200756
C	-3.379257	1.947604	-1.079340
H	-2.715853	2.839500	-1.135029
H	-3.785733	1.750099	-2.099175
H	-4.239196	2.215500	-0.416275
C	-4.765856	-1.522688	-2.755061
H	-4.372739	-2.160308	-3.579691
H	-5.281335	-2.186870	-2.025049
H	-5.532185	-0.842721	-3.195845

TS3

E= -2236.90165024 A.U.

C	-1.352000	0.823000	0.195000
N	-1.406000	2.197000	0.184000
N	-2.606000	0.434000	0.603000
C	-2.665000	2.650000	0.578000
C	-3.422000	1.539000	0.841000
C	-0.318000	3.084000	-0.177000
C	-3.041000	-0.939000	0.763000
H	-2.903000	3.717000	0.630000
H	-4.459000	1.431000	1.174000
C	-0.209000	3.504000	-1.529000
C	0.829000	4.404000	-1.845000
C	1.711000	4.868000	-0.859000
C	1.580000	4.430000	0.467000
C	0.566000	3.525000	0.842000
C	-1.145000	2.999000	-2.627000
H	0.947000	4.747000	-2.885000
H	2.514000	5.573000	-1.128000
H	2.286000	4.792000	1.230000
C	0.439000	3.074000	2.297000
C	-2.900000	-1.554000	2.035000
C	-3.363000	-2.879000	2.166000

C	-3.938000	-3.559000	1.083000
C	-4.062000	-2.925000	-0.161000
C	-3.618000	-1.601000	-0.353000
C	-2.283000	-0.839000	3.237000
H	-3.265000	-3.390000	3.137000
H	-4.291000	-4.595000	1.210000
H	-4.509000	-3.472000	-1.007000
C	-3.739000	-0.945000	-1.729000
H	-1.940000	2.394000	-2.142000
C	-0.397000	2.064000	-3.601000
C	-1.845000	4.155000	-3.372000
H	-0.286000	2.233000	2.324000
C	1.772000	2.533000	2.852000
C	-0.132000	4.206000	3.180000
H	-1.958000	0.168000	2.902000
C	-3.323000	-0.637000	4.361000
C	-1.025000	-1.571000	3.751000
H	-3.391000	0.105000	-1.633000
C	-2.813000	-1.637000	-2.752000
C	-5.201000	-0.901000	-2.220000
H	-1.094000	1.654000	-4.362000
H	0.410000	2.604000	-4.141000
H	0.063000	1.213000	-3.059000
H	-2.570000	3.758000	-4.113000
H	-2.400000	4.817000	-2.674000
H	-1.121000	4.788000	-3.927000
H	1.617000	2.110000	3.867000
H	2.183000	1.738000	2.194000
H	2.532000	3.337000	2.947000
H	-0.259000	3.858000	4.226000
H	0.551000	5.083000	3.199000
H	-1.119000	4.561000	2.816000
H	-2.879000	-0.063000	5.203000
H	-4.215000	-0.082000	4.003000
H	-3.675000	-1.608000	4.770000
H	-0.581000	-1.014000	4.603000
H	-1.269000	-2.592000	4.116000
H	-0.252000	-1.658000	2.959000
H	-2.864000	-1.121000	-3.734000
H	-1.758000	-1.624000	-2.408000
H	-3.107000	-2.696000	-2.913000
H	-5.269000	-0.368000	-3.191000
H	-5.614000	-1.920000	-2.374000
H	-5.862000	-0.379000	-1.497000
Au	0.195000	-0.388000	-0.225000
O	1.824000	-1.445000	1.678000
H	2.264000	-1.419000	2.554000
Si	3.108000	-1.178000	0.518000
O	3.072000	0.522000	0.332000
O	4.281000	-1.547000	-0.730000

O	4.194000	-1.589000	1.830000
C	1.675000	-1.874000	-0.894000
C	1.074000	-3.144000	-0.616000
C	1.841000	-1.552000	-2.283000
C	0.689000	-4.033000	-1.637000
H	0.926000	-3.423000	0.440000
C	1.439000	-2.424000	-3.306000
H	2.329000	-0.600000	-2.548000
C	0.867000	-3.671000	-2.983000
H	0.245000	-5.009000	-1.381000
H	1.577000	-2.139000	-4.362000
H	0.565000	-4.363000	-3.786000
C	5.589000	-1.428000	1.880000
H	6.048000	-1.304000	0.872000
H	5.871000	-0.533000	2.489000
H	6.065000	-2.314000	2.365000
C	4.115000	1.306000	-0.213000
H	3.697000	2.292000	-0.516000
H	4.924000	1.497000	0.533000
H	4.584000	0.826000	-1.101000
C	4.689000	-2.845000	-1.070000
H	3.867000	-3.439000	-1.547000
H	5.521000	-2.788000	-1.808000
H	5.056000	-3.431000	-0.192000

TS4

E= -2121.23554814 A.U.

C	-0.853997	1.085824	0.303195
N	-2.138872	1.046018	0.788016
N	-0.427293	2.367778	0.551170
C	-2.508449	2.280854	1.320148
C	-1.430584	3.114279	1.169658
C	-2.983560	-0.130701	0.779274
C	0.901041	2.860526	0.251634
H	-3.497349	2.448418	1.757320
H	-1.283114	4.162180	1.447262
C	-2.980420	-0.972976	1.922934
C	-3.796859	-2.122820	1.873758
C	-4.577001	-2.415708	0.747174
C	-4.566262	-1.557427	-0.361529
C	-3.771201	-0.393303	-0.373296
C	-2.161290	-0.677020	3.180408
H	-3.815903	-2.805149	2.738279
H	-5.202015	-3.322966	0.733091
H	-5.185384	-1.798620	-1.240438
C	-3.761269	0.513443	-1.604297
C	1.164893	3.356788	-1.052052
C	2.477856	3.793043	-1.323704
C	3.476195	3.742404	-0.340094
C	3.178001	3.264877	0.943404
C	1.883306	2.815034	1.276137

C	0.085650	3.452181	-2.131693
H	2.722055	4.178935	-2.326252
H	4.497180	4.082784	-0.575763
H	3.967778	3.234513	1.710340
C	1.577093	2.353414	2.702005
H	-1.593849	0.260690	3.005046
C	-1.122353	-1.780588	3.472286
C	-3.090777	-0.428161	4.390098
H	-3.140759	1.402032	-1.364876
C	-3.092969	-0.191585	-2.803447
C	-5.174085	1.024269	-1.957281
H	-0.838686	2.991594	-1.725199
C	-0.241614	4.926053	-2.460154
C	0.464966	2.659980	-3.398710
H	0.537977	1.962697	2.715574
C	2.491490	1.201115	3.166737
C	1.628832	3.556112	3.673034
H	-0.594449	-1.553955	4.422909
H	-1.605278	-2.773879	3.592636
H	-0.348463	-1.842166	2.675645
H	-2.493972	-0.148633	5.283477
H	-3.818920	0.387581	4.195470
H	-3.671411	-1.338155	4.652008
H	-3.032914	0.493312	-3.675563
H	-2.063384	-0.515836	-2.543505
H	-3.668528	-1.088313	-3.116897
H	-5.129188	1.724588	-2.817778
H	-5.853445	0.193765	-2.244260
H	-5.641720	1.559692	-1.104986
H	-1.062803	4.987518	-3.205183
H	-0.558258	5.486064	-1.555786
H	0.638031	5.452686	-2.887619
H	-0.352186	2.714449	-4.148528
H	1.381827	3.065272	-3.876923
H	0.642115	1.591486	-3.159037
H	2.252685	0.939067	4.219465
H	2.337251	0.288747	2.547302
H	3.563595	1.492691	3.139841
H	1.354193	3.236654	4.700281
H	2.648974	3.993618	3.720851
H	0.933978	4.367646	3.369329
Au	0.217717	-0.438183	-0.442487
O	1.537827	-1.431422	1.459826
C	1.461438	-2.208078	-1.307886
C	2.012033	-1.320457	-2.292500
C	0.373890	-3.042776	-1.737280
C	1.543246	-1.311159	-3.622552
H	2.869692	-0.687729	-2.012189
C	-0.089720	-3.028540	-3.061788
H	-0.068217	-3.741611	-1.008158

C	0.507429	-2.175759	-4.012053
H	2.009688	-0.639311	-4.360947
H	-0.910945	-3.699206	-3.362079
H	0.156521	-2.181964	-5.056623
Si	2.239180	-2.452046	0.434891
O	3.893052	-2.244169	0.117866
O	1.988020	-4.072663	0.856875
C	2.650441	-5.163676	0.249415
H	2.669031	-6.021814	0.957303
H	2.125239	-5.505136	-0.676304
H	3.701498	-4.921347	-0.031594
C	4.843975	-1.975840	1.132390
H	5.826632	-1.764982	0.656368
H	4.561423	-1.093146	1.751028
H	4.981376	-2.841925	1.822569

3D Structures:

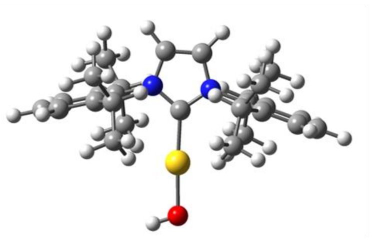

1

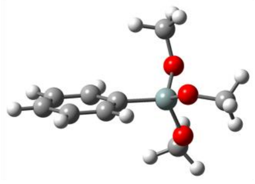

$PhSi(OMe)_3$

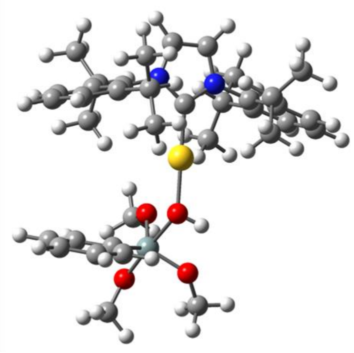

4

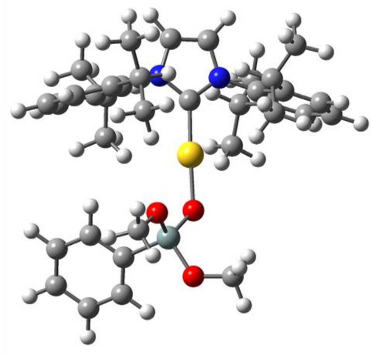

5

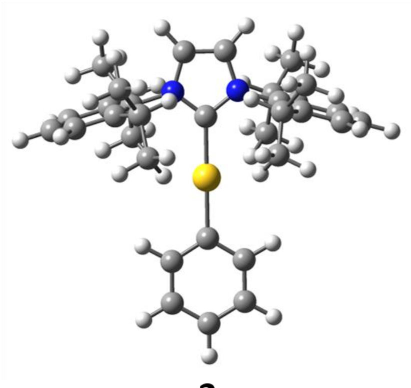

3

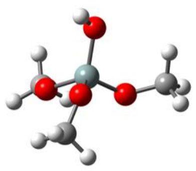

$Si(OMe)_3OH$

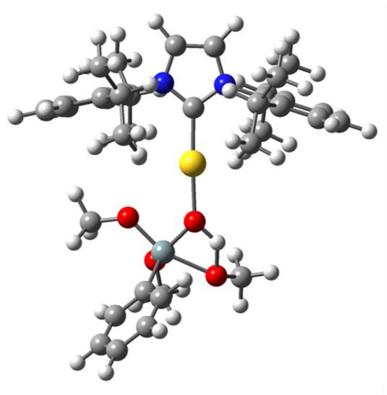

TS2

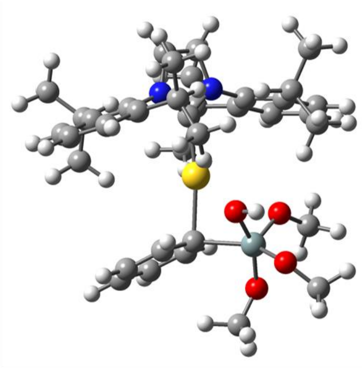

TS3

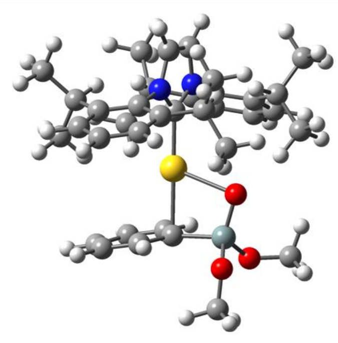

TS4

---

[i] Gaussian 09, Revision D.01, M. J. Frisch, G. W. Trucks, H. B. Schlegel, G. E. Scuseria, M. A. Robb, J. R. Cheeseman, G. Scalmani, V. Barone, B. Mennucci, G. A. Petersson, H. Nakatsuji, M. Caricato, X. Li, H. P. Hratchian, A. F. Izmaylov, J. Bloino, G. Zheng, J. L. Sonnenberg, M. Hada, M. Ehara, K. Toyota, R. Fukuda, J. Hasegawa, M. Ishida, T. Nakajima, Y. Honda, O. Kitao, H. Nakai, T. Vreven, J. A. Montgomery, Jr., J. E. Peralta, F. Ogliaro, M. Bearpark, J. J. Heyd, E. Brothers, K. N. Kudin, V. N. Staroverov, R. Kobayashi, J. Normand, K. Raghavachari, A. Rendell, J. C. Burant, S. S. Iyengar, J. Tomasi, M. Cossi, N. Rega, J. M. Millam, M. Klene, J. E. Knox, J. B. Cross, V. Bakken, C. Adamo, J. Jaramillo, R. Gomperts, R. E. Stratmann, O. Yazyev, A. J. Austin, R. Cammi, C. Pomelli, J. W. Ochterski, R. L. Martin, K. Morokuma, V. G. Zakrzewski, G. A. Voth, P. Salvador, J. J. Dannenberg, S. Dapprich, A. D. Daniels, Ö. Farkas, J. B. Foresman, J. V. Ortiz, J. Cioslowski, D. J. Fox, Gaussian, Inc., Wallingford CT, 2009.

[ii] (a) A. D. Becke, Phys. Rev. A 1988, 38, 3098-3100. (b) J. P. Perdew, Phys. Rev. B 1986, 33, 8822-8824. (c) J. P. Perdew, Phys. Rev. B 1986, 34, 7406-7406.

[iii] F. Weigend, R. Ahlrichs, Phys. Chem. Chem. Phys. 2005, 7, 3297-3305.

[iv] (a) U. Haeusermann, M. Dolg, H. Stoll, H. Preuss, Mol. Phys. 1993, 78, 1211-1224. (b) W. Kuechle, M. Dolg, H. Stoll, H. Preuss, J. Chem. Phys. 1994, 100, 7535-7542. (c) Leininger, T.; Nicklass, A.; Stoll, H.; Dolg, M.; Schwerdtfeger, P. J. Chem. Phys. 1996, 105, 1052-1059.

[v] (a) V. Barone, M. Cossi, J. Phys. Chem. A 1998, 102, 1995-2001. (b) J. Tomasi, M. Persico, Chem. Rev. 1994, 94, 2027-2094
